# A Novel Preparation Technique for Human Nasal Respiratory Mucosa to Disclose Its Glycosylation Pattern for Bioadhesive Drug Delivery

**DOI:** 10.3390/pharmaceutics15030973

**Published:** 2023-03-17

**Authors:** Julia Clara Gausterer, Michael Schlager, Navid Ahmadi, Michael Nieratschker, Valerie Dahm, Michael Wirth, Christoph Arnoldner, Clemens Honeder, Franz Gabor

**Affiliations:** 1 Division of Pharmaceutical Technology and Biopharmaceutics, Department of Pharmaceutical Sciences, University of Vienna, Josef-Holaubek-Platz 2, 1090 Vienna, Austria; 2 Department of Otorhinolaryngology-Head and Neck Surgery, Medical University of Vienna, Währinger Gürtel 18-20, 1090 Vienna, Austria

**Keywords:** lectins, WGA, mucoadhesion, intranasal drug delivery, glycotargeting

## Abstract

To shed some light on glycotargeting as a potential strategy for nasal drug delivery, a reliable preparation method for human nasal mucosa samples and a tool to investigate the carbohydrate building blocks of the glycocalyx of the respiratory epithelium are required. Applying a simple experimental setup in a 96-well plate format together with a panel of six fluorescein-labeled lectins with different carbohydrate specificities allowed for the detection and quantification of accessible carbohydrates in the mucosa. As confirmed by binding experiments at 4 °C, both quantitatively by fluorimetry and qualitatively by microscopy, the binding of wheat germ agglutinin exceeded that of the others by 150% on average, indicating a high content of N-acetyl-D-glucosamine and sialic acid. Providing energy by raising the temperature to 37 °C revealed uptake of the carbohydrate-bound lectin into the cell. Moreover, repeated washing steps during the assay gave a slight hint as to the influence of mucus renewal on bioadhesive drug delivery. All in all, the experimental setup reported here for the first time is not only a suitable approach to estimating the basics and potential of nasal lectin-mediated drug delivery but also meets the needs for answering a broad variety of scientific questions involving the use of ex vivo tissue samples.

## 1. Introduction

In times of stagnant numbers of new drug approvals coupled with ever-rising costs, the aspect of rational drug targeting applications for existing active substances attracts more and more interest. Whether the goal is a reduced side-effect profile, an increased concentration of the active pharmaceutical ingredient at the target site, or the exploration of new strategies to treat common illnesses, highly specific targeting methods for known active substances emerge as a viable complement to the discovery of new drug substances. Lectins, a ubiquitous and heterogeneous group of glycoproteins, share the ability to selectively and stereospecifically bind specific carbohydrate structures [1,2] and therefore open an interesting possibility for use in targeted drug delivery to the glycocalyx of cell membranes. The carbohydrate composition of the glycocalyx was shown to be subject to changes due to pathologic conditions like sepsis [3], gastrointestinal diseases [4], inflammatory conditions [5], and especially tumour development [6] as well as progression [7,8]. As alterations in the glycosylation profile of cancer cells were shown to affect the binding of nanoparticulate drug delivery systems compared to benign cells [9], it seems like a reasonable approach to take advantage of the highly specific carbohydrate recognition properties of plant lectins as a tool to differentiate between healthy and pathological tissue by applying lectin-modified drug delivery systems. Therefore, knowledge about the tissue-specific carbohydrate composition and alterations under pathologic conditions is crucial for the development of lectin-mediated glycotargeting strategies like lectin-functionalised drug molecules [10,11] and drug delivery systems [12,13,14,15]. The concept of plant lectin-based targeting was already investigated in the past for several different tissue types, such as the middle ear mucosa [16], the urothelium [17], and the intestinal epithelium [18], with the aim of serving as a basis for bioadhesive drug targeting. Bioadhesive nasal drug delivery systems have been shown to increase residence time at the nasal mucosa by avoiding rapid clearance [19], which is crucial for successful intranasal drug delivery. As the nose was evolutionary optimized to protect the lower respiratory tract from airborne exogenous noxious substances [20,21], 80–90% of the human nasal cavity is lined with nasal respiratory epithelium [22,23], which is coated with a continuous, biphasic 5–15 µM thick layer of mucus [20,24,25]. On the one hand, the biphasic mucus layer is a diffusion barrier for particulate drug delivery systems, limiting the rate of drug transport to the respiratory epithelium [26]. On the other hand, it is responsible for the short residence time of intranasally applied formulations, as they are removed from the mucosa via mucociliary clearance with an average speed of 6 mm/min [27]. Despite these difficulties, the respiratory epithelium provides—due to its large area (150 cm^2^) and high vascularisation—desirable features for systemic intranasal drug delivery [22,28]. Rapid drug absorption and onset of action and increased bioavailability by avoiding the hepatic first-pass metabolism render nasal drug delivery a non-invasive, easily accessible, and patient-friendly route of administration suitable for self-medication [29].

This work is aimed at elucidating the glycosylation pattern and the lectin binding capacities of the healthy human nasal mucosa by using an array of six different fluorescein-labeled plant lectins and applying a novel and simple technique for sample preparation. In this context, the extent of active transport of tissue-associated lectins into nasal epithelial cells was investigated as well. Additionally, a microplate-formatted experimental setup is presented that enables reliable and reproducible fluorometric measurements of ex vivo tissue samples.

## 2. Materials and Methods

### 2.1. Materials

The six fluorescein-labeled lectins Galanthus nivalis lectin (GNL; n = 8), Lens culinaris agglutinin (LCA; n = 5), Peanut agglutinin (PNA; n = 8), Solanum tuberosum lectin (STL; n = 8), Ulex europaeus agglutinin 1 (UEA I; n = 8), and Wheat germ agglutinin (WGA; n = 8) were purchased from Vector Laboratories (Burlingame, CA, USA). The lectin working solutions were prepared by diluting the stock solutions with simulated nasal electrolyte solution (SNES) to a final lectin concentration of 500 nmol/L. The lectin concentration was chosen based on a study by Engleder, Demmerer, Wang, Honeder, Zhu, Studenik, Wirth, Arnoldner, and Gabor [16] in a guinea pig model. The SNES contained 7.45 mg/mL of sodium chloride, 1.29 mg/mL of potassium chloride, and 0.32 mg/mL of calcium chloride dihydrate in distilled water [30]. All chemicals used for the preparation of SNES were of analytical grade and purchased from Carl Roth GmbH + Co. KG (Karlsruhe, Germany). This solution also served as a washing solution in the experiment. A solution of fluorescein isothiocyanate-conjugated bovine serum albumin (F-BSA; F/P ratio = 7–12; Sigma-Aldrich Corporation, St. Louis, MO, USA) in SNES (500 nmol/L) was used to rule out any non-specific protein binding. For lectin properties (source, molecular weight, binding motif, and molar fluorescein/protein (F/P) ratio), see Table 1.

### 2.2. Preparation of Human Nasal Mucosa Samples and Experimental Setup

The nasal mucosa tissue samples were collected during standard turbinoplasty surgeries at the Department of Otorhinolaryngology, Medical University of Vienna, Vienna, Austria. The study was approved by the ethics committee of the Medical University of Vienna (Nr. 1446/2016), and each patient provided written informed consent to the histological analysis of the routinely resected mucosa. Inclusion criteria were defined as a patient age of 18 years or older, an indication for turbinoplasty with or without septo(rhino)plasty, and the absence of acute or chronic rhinosinusitis. Samples were stored at 4 °C in a tightly closed container in a moist atmosphere to prevent drying of the specimen and were used for binding studies within two to three hours after collection. As several authors noticed that different tissue fixation techniques can impact lectin binding to the glycocalyx [33,34,35], any unnecessary manipulations of the glycocalyx were avoided by using unfixed, native tissue samples to guarantee reliable fluorometric quantification of lectin binding that reflects the intrinsic glycosylation pattern of the human nasal mucosa.

Prior to the experiments, silicone platelets were prepared by spreading liquid silicone (Silicone Rubber Adhesive Sealant, Momentive Performance Materials GmbH, Leverkusen, Germany) on the backside of the wells of a 96-well microplate and using them as a mold for the preparation of approximately 1 mm thick silicone discs. After curing for at least 16 h at room temperature, silicone discs with a 5 mm diameter were punched out using a hollow punch corresponding to the size of the mucosa punches.

**Figure 1:**
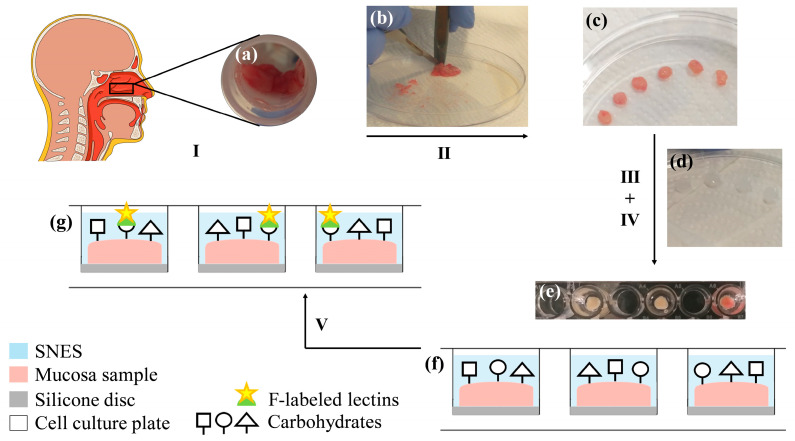
Experimental setup for human nasal ex vivo samples. I. Collection of healthy human nasal mucosa tissue (**a**) during routine turbinoplasty surgery. II. Using a punch and scalpel (**b**) samples are cut into round pieces of 5 mm in diameter (**c**). III. Specimens are fixed on a silicone disc (**d**) with the mucosal side facing upward, using acrylic glue. IV. Sample discs are placed into the well of a 96-well cell culture plate and attached to the bottom with acrylic glue. To prevent fluorescent probes in neighboring wells from mutually interfering with their fluorometric measurements, every other well is left empty (**e**). The sample surface is covered with simulated nasal electrolyte solution (SNES) (**e**,**f**). V. Samples are incubated with fluorescein (F)-labeled lectins to elucidate the glycosylation pattern and the lectin binding capacities of the healthy human nasal mucosa (**g**).

Similarly, human nasal mucosa samples were cut into round pieces of 5 mm in diameter using the hollow punch and a scalpel. Specimens were carefully patted dry on the bottom with a fiber-free tissue and fixed on the silicone disc with the mucosal face upwards using acrylic glue (Pattex Power Easy Gel, Henkel AG & Co. KGaA, Düsseldorf, Germany). After hardening, the sample disc was transferred into the well of a 96-well cell culture plate (Greiner Bio-One, Kremsmünster, Austria) and attached to the bottom with acrylic glue, using forceps and gel loading tips for positioning (Figure 1). Prior to the experiments, the silicone discs and the acrylic glue were tested for any detrimental effects on polystyrene microplates, for autofluorescence, and for any potential adhesion of fluorescent lectins to exclude any potential interferences and any falsification of the fluorometric quantification (see Section 3.1).

### 2.3. Fluorimetric Quantification of the Lectin Binding Capacity of the Human Nasal Mucosa

The mucosa samples were washed three times with 200 µL of SNES, immersed in 200 µL of SNES, and kept on ice for 30 min. After equilibration, the specimens were covered with 200 µL of fresh SNES, and the autofluorescence of the nasal mucosa surface tissue was recorded, serving as a baseline for subsequent calculations. All fluorometric data were collected with an Infinite M200 PRO instrument (Tecan Group Ltd., Männedorf, Switzerland) at 485 nm/525 nm (exc./em.) and a gain of 60 (top reading mode).

The samples were then incubated on ice with 200 µL of a 500 nM lectin solution in SNES for 30 min at 4 °C. After discarding the lectin solution, the specimens were washed three times with 200 µL of ice-cold SNES. After addition of 200 µL of fresh SNES, the relative fluorescence intensity (RFI) was read as described above (4 °C).

Subsequently, samples were incubated at 37 °C for 60 min to allow for the uptake of energy and thus active transport of membrane-bound lectins into the cell. After stopping energy-consuming processes by putting the microplate on ice, the RFI was read again (37 °C/R1). Subsequently, the specimens were washed again three times as described above, and the RFI was recorded (37 °C/R2).

To recognize any contribution of non-specific protein binding to the nasal mucosa, control samples were incubated with 500 nM of F-BSA in SNES according to the protocol described above. Samples were protected from light during the entire experiment to prevent photobleaching of fluorescein-labeled lectins, and the microplates were covered with a sealing tape to minimize evaporation of SNES and thus related fluctuations in concentration.

### 2.4. Microscopical Evaluation of Lectin Binding

For qualitative confirmation of the results, lectin-labeled tissue was analyzed by confocal microscopy. Mucosal tissue was carefully dissected and prepared as whole mounts. After three washing steps with 200 µL of SNES each, the specimens were equilibrated in ice-cold SNES for 30 min. The tissue was then incubated in 200 µL of 500 nM fluorescein-labeled lectin (F-PNA, F-UEA I, or F-WGA) or F-BSA (negative control) for 60 min at 4 °C. After three additional washing steps with 200 µL of SNES each, the labeled tissue was fixed for two hours in 4% aqueous paraformaldehyde. Subsequently, the specimens were washed twice, mounted on glass cover slips, and embedded in ProLong™ Gold Antifade Mountant (Thermo Fisher Scientific, Vienna, Austria) for further imaging.

Confocal images were acquired with a NIKON TI Eclipse confocal microscope at 20× magnification (0.5 numerical aperture) at 488/525 nm (exc./em.). The laser power for each individual lectin was set according to the differences in their F/P ratios (F-WGA 0.9, F-UEA I 0.765, F-PNA 0.729, and F-BSA 0.324).

### 2.5. Calculations

The relative fluorescence intensity (RFI) at 4 °C and 37 °C was calculated by subtracting the samples’ baseline autofluorescence intensities. These values were then used to calculate the molar concentrations of the lectins (4 °C, 37 °C/R1, 37 °C/R2) based on a separate, lectin- and temperature-specific standard curve. Molar concentration values (nM) for 4 °C, 37 °C/R1 (before washing), and 37 °C/R2 (after washing) are provided in Table S1. The results obtained after incubation at 4 °C depict the lectin binding capacity of the human nasal mucosa in the absence of active, energy-driven transport processes, whereas values after incubation at 37 °C represent both mucosa-surface bound as well as intracellular amounts of lectin before (37 °C/R1) and after washing (37 °C/R2).

The relative uptake (%), describing the membrane-bound amount of lectin at 4 °C that became internalized after rising the temperature to 37 °C, was evaluated according to:

(1) $$\mathrm{Internalisation}\;[\%]=1\;-\;\left(\frac{37\;{}^{\circ}C/R1}{4\;{}^{\circ}C}\right)*100$$

The molar lectin uptake was calculated according to the following equation:

(2) Internalisation [nM]=4 °C − 37 °C/R1

The proportional reduction of mucosa-associated lectin, stating the fraction (%) of 37 °C/R1 being removed by washing, was calculated as follows:

(3) $$\mathrm{Reduction}\;[\%]=1\;-\;\left(\frac{37\;{}^{\circ}C/R2}{37\;{}^{\circ}C/R1}\right)*100$$

### 2.6. Statistical Analysis

Data are stated as mean ± SD. Error bars represent SD. Data analysis was performed using GraphPad Prism version 9.4.1 software (GraphPad software, San Diego, CA, USA). Differences in the lectin binding capacity of the nasal mucosa at 4 °C and 37 °C were statistically analysed using a Welch’s one-way analysis of variance (ANOVA) test followed by a Dunnett’s T3 test for multiple comparisons. Relative (%) and absolute (nM) internalisation of previously mucosa-bound lectin and the proportional reduction of lectin binding (%) due to washing were statistically evaluated by an ANOVA test and a Tukey’s multiple comparisons test. The influence of temperature increase on lectin binding was statistically assessed by a two-tailed paired *t*-test. All statistical tests were two-sided, and results were considered statistically significant if *p* ≤ 0.05.

## 3. Results and Discussion

### 3.1. Considerations for the Experimental Setup

In order to shed some light on the glycosylation pattern of human mucosa, there are special demands on the experimental methodology as well as on the materials in use. The assay design should (i) be applicable to tissue samples of various thickness and size; (ii) preserve the structural integrity of the multi-layered human mucosa as well as that of the covering glycocalyx; (iii) be carried out in a 96-well microplate format suitable for read-out in a plate reader; (iv) use materials that are easily available and cost-effective but at the same time must not interfere with fluorimetry; and (v) provide reliable and reproducible fluorescence readouts. In preliminary experiments, adhesives, silicones, and microplates from different manufacturers were tested for their suitability to optimally rule out the following issues: (i) any influence on or alterations of the nasal mucosa’s intrinsic lectin binding capacity; (ii) potential falsification of results due to unwanted adhesion of lectins to components such as cell culture plastics, silicone, and adhesives; (iii) any interference with the fluorometric detection; (iv) any corrosive interactions between adhesive and polystyrene or silicone; (v) any interaction with the aqueous environment (SNES) or film formation on the buffer surface by certain adhesives; and (vi) any encrustation of the mucosa surface by the adhesive as observed upon use of regular cyanoacrylate tissue adhesive. Special emphasis was also placed on the solid fixation of the mucosa sample within the well, ensuring its accurate positioning despite suction during washing. This could be achieved by choosing a silicone material that features adequate rigidity after curing and that serves as a mount for the tissue, preventing any deformation of the sample shape. Strong and durable adhesion to the microplate was achieved by using a fast-drying acrylic glue.

The experimental setup presented (Figure 1) complied with these requirements, yielding consistent and reproducible fluorometric read-outs with a reasonable deviation. The variance observed can be attributed to interindividual differences in lectin binding capacities and was predictable since ex vivo tissue from a wide array of different patients was used. Furthermore, this experimental setup is not limited to nasal mucosa tissue but can also be adapted to a broad variety of research questions requiring ex vivo tissue samples of certain size and thickness.

### 3.2. Lectin Binding Studies

When cell binding studies are performed at 4 °C, no energy-consuming cellular processes should take place, and therefore, active transport and uptake processes should be reduced to a minimum. This setup provides information about the native, intrinsic glycosylation pattern of the human nasal mucosa as indicated by its binding capacity for fluorescein-labeled lectins with different carbohydrate specificities.

**Figure 2:**
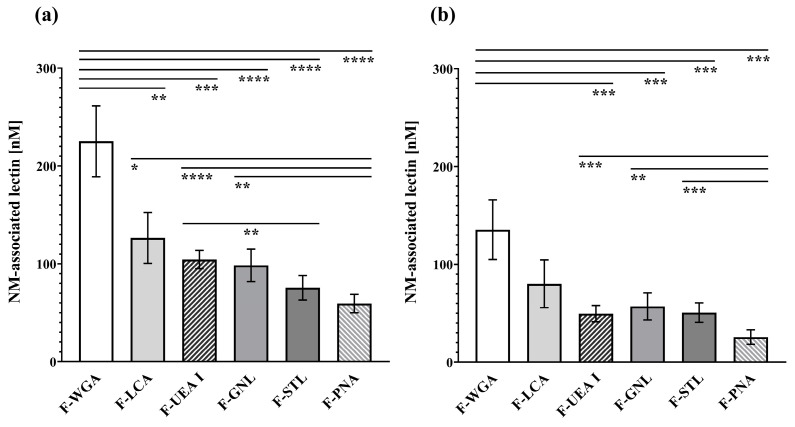
Lectin-binding capacity of the human nasal mucosa at (**a**) 4 °C and (**b**) 37 °C. Nasal mucosa (NM); Wheat germ agglutinin (WGA); Lens culinaris agglutinin (LCA); Ulex europaeus agglutinin 1 (UEA I); Galanthus nivalis lectin (GNL); Solanum tuberosum lectin (STL); and Peanut agglutinin (PNA). * *p* ≤ 0.05; ** *p* ≤ 0.01; *** *p* ≤ 0.001; and **** *p* ≤ 0.0001.

Among all lectins investigated at 4 °C, F-WGA showed the strongest mucosa-adhesive properties (225.2 ± 36.26 nM), corresponding to 45.04 ± 7.25% of lectin molecules bound to the nasal mucosa tissue. F-LCA, although being the second in line, exhibited a significantly lower adherence to the human nasal mucosa (126.5 ± 26.05 nM), resulting in a 44% reduction in lectin binding compared to F-WGA. Nasal mucosa binding at 4 °C did not significantly differ between F-LCA (126.50 ± 26.05 nM), F-UEA I (104.30 ± 9.38 nM), and F-GNL (98.45 ± 16.57 nM), with F-UEA I exhibiting only a slightly higher mean mucosa-associated lectin concentration compared to F-GNL. In contrast, the adhesion of F-STL to the nasal mucosa was significantly lower than that of F-UEA I (75.47 ± 12.55 nM). Furthermore, the mucosa-bound amounts of F-PNA (59.39 ± 9.46 nM) were significantly lower than those of the other lectins except for F-STL, which occupies the penultimate position upon ranking the lectins based on their binding capacities at 4 °C. In summary, lectin association with the human nasal mucosa at 4 °C followed the order: WGA > LCA > UEA I > GNL > STL >PNA (Figure 2a, Tables S1 and S2).

Subsequent incubation of nasal mucosa samples at 37 °C enables energy-consuming cellular transport processes. To allow a reliable statement on the further fate of the lectin corona bound at 4 °C, the human nasal mucosa samples were incubated at 37 °C, and SNES was changed prior to measurement to mimic the low retention time of mucus (37 °C/R2).

At 37 °C, in accordance with data at 4 °C, F-WGA also exhibited the highest association capacity with the nasal mucosa (135.40 ± 30.55 nM) and significantly exceeded the cell-associated amounts of the other lectins except for F-LCA (80.13 ± 24.37 nM). In contrast, the F-PNA binding capacity at 37 °C lagged far behind and ranked last among the lectins investigated with a concentration of 25.57 ± 7.34 nM. Lectin association to the nasal mucosa did not significantly differ between F-LCA, F-GNL (56.97 ± 13.93 nM), F-STL (50.63 ± 9.93 nM), and F-UEA I (49.59 ± 8.35 nM). In general, the lectin association data of the human nasal mucosa at 37 °C and at 4 °C followed the same trend with slight variations in the “midfield”. F-GNL, F-STL, and F-UEA I switched places compared to the 4 °C data, resulting in the order: WGA > LCA > GNL > STL > UEA I > PNA (Figure 2b, Tables S1 and S2).

Although the temperature increase to 37 °C provoked a change in the order of lectin binding capacity of F-UEA I, F-GNL, and F-STL (4 °C: UEA I > GNL > STL; 37 °C: GNL > STL > UEA I), the differences between lectins lack statistical significance (Figure 2b, Table S2). Therefore, we can assume that the glycosylation pattern of the human nasal mucosa does not differ between 4 °C and 37 °C, suggesting that a temperature increase does not influence the accessibility of the carbohydrate structures. In binding studies at both temperature levels, WGA emerged as the lectin with the highest binding potential to healthy human nasal mucosa. Thus, (terminal) N-acetylglucosamine residues and sialylated glycans play a dominant role in the glycocalyx of healthy human nasal mucosa due to their high accessibility and/or prevalence.

Furthermore, the nasal mucosa possesses a significant binding capacity for LCA and UEA I, referring to the presence of two regioisomeric fucose structures: α1-2 linked fucose (UEA I) and α1-6 linked fucose, the latter being part of the more complex binding motif of LCA [32]. Furthermore, terminal α1-3 linked mannose structures (GNL) seem to be present.

Linear poly-N-acetyl-lactosamine (STL) and Galβ1-3GalNAc (i.e., T antigen; PNA) structures seem to play a marginal role as a component of the human nasal mucosa glycocalyx. The low adhesion of PNA in comparison to WGA strongly supports the theory of a high degree of sialylation, as PNA only binds to nonsialylated Galβ1-3GalNAcα1-Ser/Thr in O-glycans [32].

The high degree of sialylation goes hand in hand with negative net charges [36], suggesting that positively charged drug delivery systems would be promoted via electrostatic interactions [37]. Using porcine nasal epithelium, Clementino et al. [38] observed that positively charged hybrid lecithin/chitosan nanoparticles exhibited a pronounced mucoadhesive effect, resulting in a significant increase in the mucosal mean residence time.

The results presented roughly match with data published on the glycoconjugate expression in the glycocalyx of the normal human inferior turbinate mucosa by Berger et al. [39]. Their work, however, did not reveal any significant differences in the concentrations of N-acetylglucosamine, fucose, mannose, and sialic acid in the glycocalyx [39]. Presumably, this divergence can be attributed to fundamental differences in the experimental methodology, as lectin binding was microscopically evaluated by employing a semiquantitative staining scoring system. In contrast, this work relies on the objective, fluorometric quantification of the lectin binding capacity of the human nasal mucosa surface using whole mount-tissue samples. This approach additionally provides information about lectin uptake that is indicative of the utility of an individual lectin for intranasal drug delivery systems.

### 3.3. Microscopical Visualisation of Lectin Binding Capacities

To visualise the lectin binding pattern of the human nasal mucosa and verify the results of the binding studies qualitatively, three lectins exhibiting high (F-WGA), moderate (F-UEA I), and low (F-PNA) mucosa binding capacities in previous binding studies at 4 °C were chosen. F-BSA served as a negative control to cover any unspecific protein binding.

**Figure 3:**
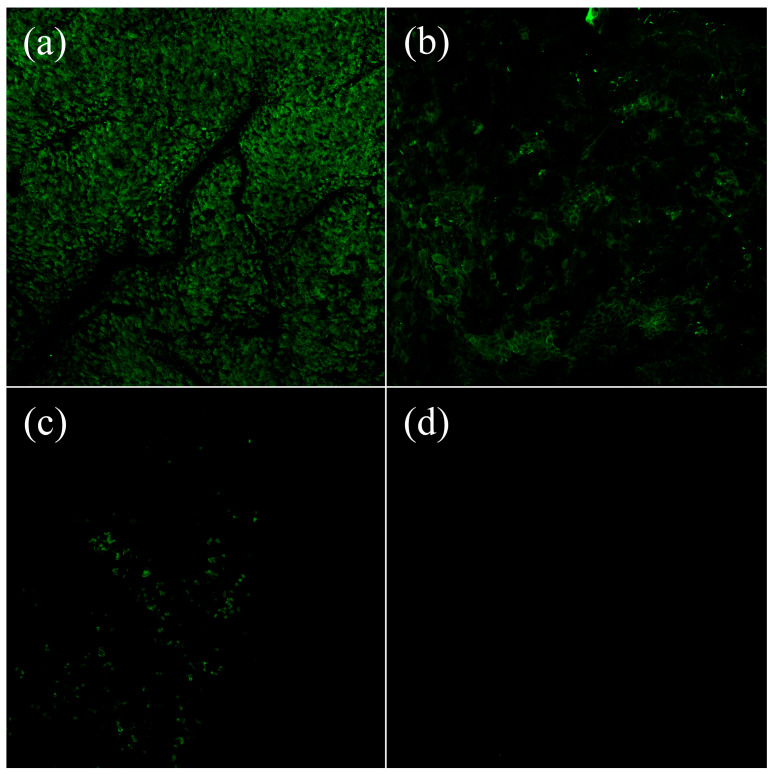
Microscopical visualisation of the lectin binding patterns of (**a**) F-WGA, (**b**) F-UEA I, (**c**) F-PNA, and (**d**) F-BSA after incubation at 4 °C.

F-WGA showed a bright, extensive, and uniform binding pattern throughout the sample surface, and the morphological structures of the mucosa surface were distinct and clearly visible. In contrast, incubation with F-UEA I revealed a noticeably weaker, yet evenly distributed staining pattern, indicating strongly reduced binding compared to F-WGA. F-PNA was detected in a very few scattered and isolated spots of the nasal mucosa, associated with a very weak fluorescence signal intensity (Figure 3).

The lack of any staining with F-BSA confirmed that unspecific proteins did not interfere with histochemical analysis. All in all, fluorescence microscopy confirms the quantitative data. F-WGA shows the highest binding capacity to human nasal mucosa, followed by F-UEA I and F-PNA. Incubation with F-BSA excluded any contribution of nonspecific protein-protein interactions so that the results reflect the glycosylation pattern of the human nasal mucosa.

### 3.4. Internalisation and (Intra)Cellular Fate of Lectins

**Figure 4:**
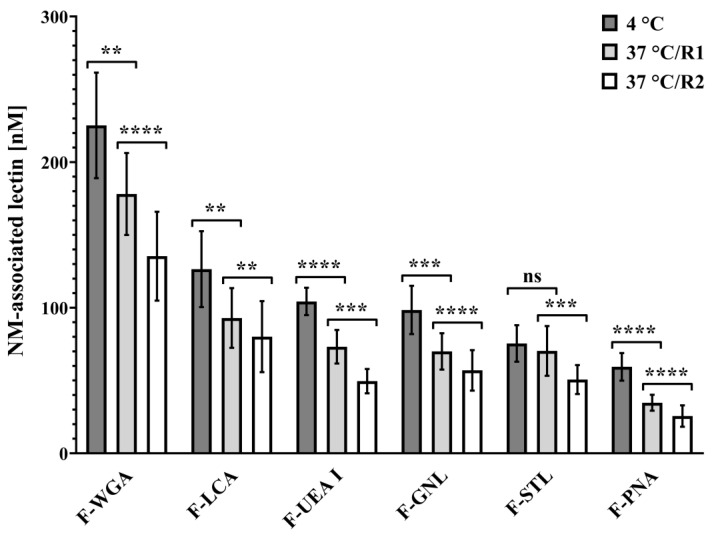
Influence of temperature increase and washing procedure on lectin-cell association. Values obtained at 37 °C were measured before (37 °C/R1) and after washing (37 °C/R2). ns (not significant) *p* > 0.05; ** *p* ≤ 0.01; *** *p* ≤ 0.001; and **** *p* ≤ 0.0001.

The mucosa-associated fluorescence intensity of all lectins investigated except for F-STL significantly decreased when the temperature was increased from 4 °C to 37 °C to provide energy for active transport processes (Figure 4, Table S1).

This decrease in fluorescence intensity is primarily due to uptake into the acidic lysosomal compartments of the cells [40,41]. The acidic environment there quenches the quantum yield of the pH-sensitive fluorescein label of the lectins. Additionally, shielding of the quantum yield by the cell membrane due to internalisation as well as detachment of initially mucus-bound lectin might contribute to a negligible extent.

When interpreting the extent of lectin uptake into the cell as given by the decrease in the concentration of mucosa-associated lectin after a temperature increase from 4 °C to 37 °C, the relative lectin uptake (%) represents the actual proportion of previously cell-bound lectin that was taken up by the epithelium, and the absolute lectin uptake (nM) indicates the number of internalised lectin molecules.

**Figure 5:**
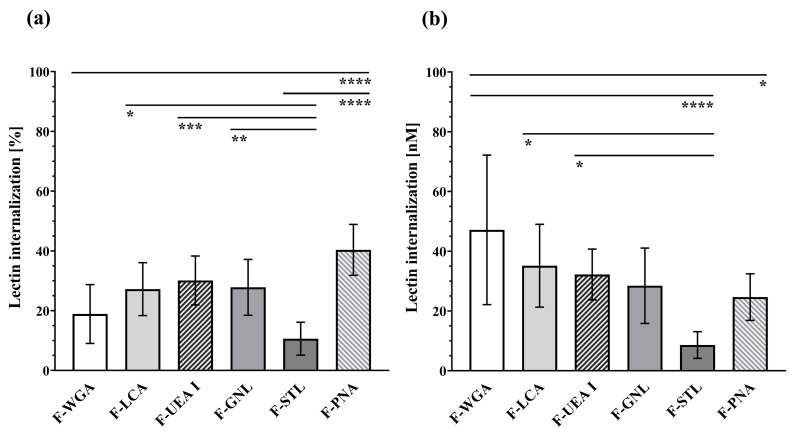
Internalisation of lectins at 37 °C. Values represent the (**a**) relative (%) and (**b**) absolute (nM) uptake of mucosa-bound lectins at 4 °C after a temperature increase to 37 °C. * *p* ≤ 0.05; ** *p* ≤ 0.01; *** *p* ≤ 0.001; and **** *p* ≤ 0.0001.

Although F-PNA possesses the lowest mucosa binding capacity at 4 °C (Figure 2a), it exhibits the highest relative uptake (40.34 ± 8.52%) of all lectins tested and significantly exceeds those of F-WGA (18.88 ± 9.85%) and F-STL. In contrast, the internalisation rate of F-STL was only 10.62 ± 5.50% at 37 °C and significantly lower than those of F-PNA, F-GNL, F-UEA I, and F-LCA. The relative internalisation of F-LCA (27.22 ± 8.86%), F-UEA I (30.12 ± 8.18%), and F-GNL (27.82 ± 9.34%) was comparable. The mean proportional internalisation rates followed the following order: PNA > UEA I > GNL > LCA > WGA > STL (Figure 5a, Table S3).

In contrast to the relative uptake data, F-WGA exhibited the highest mean absolute internalisation (47.14 ± 25.03 nM), significantly exceeding the uptake of F-STL (8.62 ± 4.47 nM) and F-PNA (24.65 ± 7.80 nM). In accordance with internalisation rate, F-STL showed the lowest absolute internalisation (8.62 ± 4.47 nM) in this experiment, being significantly lower than F-UEA I (32.22 ± 8.50 nM) and F-LCA. Statistical analysis did not reveal differences in absolute lectin uptake between F-WGA, F-LCA (35.15 ± 13.87 nM), F-UEA I (32.22 ± 8.50 nM), and F-GNL (41.47 ± 12.69 nM), which is consistent with results of relative uptake. In summary, mean absolute uptake values (nM) followed the order: WGA > LCA > UEA I > GNL > PNA > STL (Figure 5b, Table S3).

Overall, these findings suggest that although PNA exhibits the highest proportional endocytotic capacity, WGA exhibits both the highest absolute endocytotic capacity as well as the highest binding potential for the human nasal mucosa at body temperature. These beneficial properties make WGA a promising tool for surface modification of nanoparticular drug delivery systems to both increase residence time as well as drug bioavailability at the human nasal mucosa after intranasal application. This concept has already been proven successful by Gao et al. [42], when surface functionalisation of poly (ethylene glycol)/poly (lactic acid) nanoparticles with WGA led to a two-fold increase in brain uptake of the fluorescent model drug coumarin in rats, effectively improving nose-to-brain delivery after intranasal administration [42].

### 3.5. Interplay between the Mucus Layer and Cell Association

Under physiologic conditions, the surface of the respiratory epithelium is covered by the inner, watery, and low-viscous sol phase of the respiratory mucus, the periciliary layer [25,43]. By immersing the cilia and enabling their ciliary motion, the outer, more viscous gel layer is cleared off [36,44] within 15–20 min [23]. This continuous clearance of mucus requires a production of 1.5–2 L of mucus per day [20].

Due to the washing steps required by the protocol, it is assumed that most of the protective respiratory mucus layer was removed, revealing the underlying respiratory epithelium and the associated glycocalyx for incubation with fluorescein-labeled lectins. On the one hand, this represents a limitation of the assay, as the viscous, gel-like mucus layer is a major barrier to nasal drug delivery by physically impeding the cellular uptake of intranasally applied substances by the respiratory epithelium. On the other hand, this work is focused on the characterisation of the glycosylation pattern of the human nasal mucosa and thus, more specifically of the underlying epithelium.

**Figure 6:**
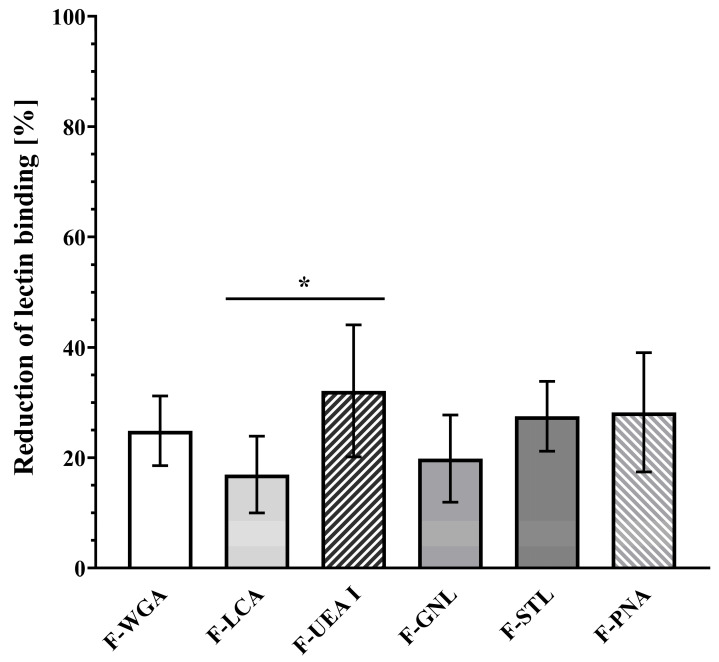
Reduction of mucosa-associated lectin by washing. The values presented were obtained at 37 °C. Statistical analysis did not reveal any significant difference between lectins (*p* > 0.05) except for F-LCA vs. F-UEA°I (*p* = 0.0463). * *p* ≤ 0.05.

Interestingly, an additional washing step decreased the association of all lectins with already “precleaned” mucosa samples (37 °C/R2, Figure 4). Statistical analysis revealed an overall mean reduction of 25.45 ± 9.63%, meaning that from 16.94 ± 6.97% (F-LCA) up to 32.11 ± 11.97% (F-UEA I) of fluorescein-labeled lectin molecules initially associated with the mucosa at 37 °C were not accessible for fluorometric quantification anymore after washing. There was no significant difference in washing-induced reduction between the lectins except for F-UEA I and F-LCA (*p* = 0.0463). The lack of statistical differences indicates that the higher binding capacity of certain lectins, e.g., WGA and LCA, can be attributed to the predominant expression of certain complementary carbohydrates and not to individual, lectin-specific differences in the stability of lectin-carbohydrate interactions (Figure 6 and Table S4).

This theory is supported by the observation that carbohydrates prevalent in the glycocalyx mostly match those commonly building up the mucus layer, e.g., sialic acid, N-acetylglucosamine, N-acetylgalactosamine, fucose, galactose, and mannose [45]. This correlation is not surprising since the glycocalyx is made up primarily of cell-associated mucins and the mucus layer is composed of secreted gel-forming mucins [36], both sharing common features such as carbohydrates [45].

Although by no means all-encompassing, the washing procedure used in this assay sheds some light on mucus loss and is an impressive hint at the great impact of mucus on nasal drug delivery. To increase the bioavailability of nasally applied drugs, delivery systems must provide both mucoadhesive and mucopermeating properties to ensure prolonged residence time and successful transmucosal transport [26] to reach therapeutically relevant drug levels in the nasal epithelium. Thus, lectin modification of drug delivery systems can not only improve association with the nasal epithelium and subsequent internalisation via lectin-carbohydrate interactions but also provide desirable mucoadhesive properties to increase the usually short residence time for successful nasal drug delivery.

## 4. Conclusions

The experimental setup reported here for the first time represents a suitable and reliable tool for the fluorometric characterisation of histochemically stained ex vivo tissue that enables reproducible quantification of cell-surface-associated fluorescent probes. The concept reported can easily be adapted to meet the needs of answering a broad variety of scientific questions involving the use of ex vivo tissue samples.

Among the lectins under investigation, WGA emerged as the lectin with both the highest binding and the highest endocytotic capacity in the human nasal respiratory epithelium. This implies that the most abundant, accessible molecules in the glycocalyx are N-acetyl-D-glucosamine and sialic acid, whereas other carbohydrates such as α-mannose and α- L-fucose seem to be less prevalent. The decrease in mucosa-associated fluorescence intensity upon increasing the temperature to 37 °C primarily indicates an energy-dependent, active internalisation of WGA. These observations suggest that lectin-grafted formulations can prolong the drug’s residence time at the nasal mucosa, increasing the drug’s bioavailability in the epithelium. The determination of the glycosylation pattern of the healthy human nasal mucosa is a first step to evaluating the potential suitability of certain lectins for improving intranasal drug delivery. Pathologic conditions like rhinitis, allergy, and sinusitis can alter the frequency of the ciliary beating, impacting mucociliary clearance [20], and mucus properties like pore size, composition, thickness, and viscoelasticity [26] represent other factors that have to be taken into account for the development of intranasal drug delivery systems. Therefore, further investigations elucidating the influence of pathologic conditions on the lectin binding and uptake of the nasal mucosa are needed in any case. Nevertheless, lectin-mediated nasal drug delivery offers a myriad of therapeutic possibilities for local, systemic, and nose-to-brain delivery of drugs via the nasal route.

## Figures and Tables

**Table 1:** Lectin characteristics.

Lectin	Source	Molecular Weight ^1^ (kDa)	Binding Motif ^2^	Molar F/P Ratio ^3^
WGA	*Triticum vulgare* (wheat germ)	36	Terminal N-acetyl-glucosamine; Sialylated glycans; Branched poly-N-acetyl-lactosamine (“I” antigen).	3.4
LCA	*Lens culinaris* (lentil)	50	Di-, triantennary Core α6-fucosylated complex-type N-glycans (but not tetra-antennary or 2,4-branched triantennary N-glycans).	3.5
UEA I	*Ulex europaeus* (gorse)	63	α1-2-linked fucose.	4.0
GNL	*Galanthus nivalis* (snowdrop)	50	Terminal α1-3- linked mannose.	4.0
STL	*Solanum tuberosum* (potato)	100	Linear poly-N-acetyl-lactosamine (“i” antigen).	3.0
PNA	*Arachis hypogaea* (peanut)	110	Core 1 (T Antigen; Galβ1-3GalNAcαSer/Thr).	4.2

^1^ from Vector Laboratories Inc. [31]; ^2^ from Cummings et al. [32]; and ^3^ according to certificates of analysis provided by the manufacturer. Wheat germ agglutinin (WGA); Lens culinaris agglutinin (LCA); Ulex europaeus agglutinin I (UEA I); Galanthus nivalis lectin (GNL); Solanum tuberosum lectin (STL); Peanut agglutinin (PNA); Galactose (Gal); N-acetylgalactosamine (GalNAc); Serin (Ser); and Threonin (Thr).

## Data Availability

The data presented in this study are available in the supplementary material.

## Supplementary Materials

The following supporting information can be downloaded at: https://www.mdpi.com/article/10.3390/pharmaceutics15030973/s1, Table S1: Concentrations (nM) of mucosa-associated lectins at 4 °C and 37 °C; Table S2: Multiple comparisons of lectin binding data at 4 °C and 37 °C; Table S3: Results of uptake studies; Table S4: Reduction of mucosa-associated lectins by washing.
