# Supplementary material for: A Novel Preparation Technique for Human Nasal Respiratory Mucosa to Disclose Its Glycosylation Pattern for Bioadhesive Drug Delivery
Source: Pharmaceutics. 2023 Mar 17;15(3):973. doi: 10.3390/pharmaceutics15030973 (PMC10052101; doi:10.3390/pharmaceutics15030973)
Supplement: Supplementary file 1 [file pharmaceutics-15-00973-s001.zip › pharmaceutics-2225732-supplementary.pdf]

Table S1. Concentrations (nM) of mucosa-associated lectins at 4 °C and 37 °C.

Lectin	Amount of lectin [nM] (mean ± SD) 4 °C	37 °C/R1	37 °C/R2	Two-tailed P value 4 °C vs. 37 °C/R1	37 °C/R1 vs. 37 °C/R2
**F-WGA**	225.20 ± 36.26	178.10 ± 28.15	135.40 ± 30.55	**0.0011**	**<0.0001**
**F-LCA**	126.50 ± 26.05	92.95 ± 20.51	80.13 ± 24.37	**0.0058**	**0.0056**
**F-UEA I**	104.30 ± 9.38	73.21 ± 11.56	49.59 ± 8.35	**<0.0001**	**0.0003**
**F-GNL**	98.45 ± 16.57	70.00 ± 12.51	56.97 ± 13.93	**0.0004**	**<0.0001**
**F-STL**	75.47 ± 12.55	70.33 ± 17.05	50.63 ± 9.93	0.1318	**0.0004**
**F-PNA**	59.39 ± 9.46	34.74 ± 5.45	25.57 ± 7.34	**<0.0001**	**<0.0001**

Values at 37 °C were obtained before washing (37 °C/R1) and after washing (37 °C/R2) of the human nasal mucosa. Statistical differences were assessed by a paired t-test. P values in bold indicate statistically significant differences between comparisons. Results were considered statistically significant if $p \leq 0.05$. Binding data is presented in Figure 2 and Figure 4; statistical significance is displayed in Figure 4.

Table S2. Multiple comparisons of lectin binding data at 4 °C and 37 °C.

Lectin		Adjusted P value 4 °C	37 °C
**F-WGA**	vs. F-LCA	**0.0019**	0.0558
	vs. F-UEA I	**0.0002**	**0.0007**
	vs. F-GNL	**< 0.0001**	**0.0008**
	vs. F-STL	**< 0.0001**	**0.0009**
	vs. F-PNA	**< 0.0001**	**0.0001**
**F-LCA**	vs F-WGA	**0.0019**	0.0558
	vs. F-UEA I	0.6639	0.2999
	vs. F-GNL	0.4948	0.6035
	vs. F-STL	0.0789	0.3398
	vs. F-PNA	**0.0236**	0.0610
**F-UEA I**	vs F-WGA	**0.0002**	**0.0007**
	vs F-LCA	0.6639	0.2999
	vs. F-GNL	0.9967	0.9349
	vs. F-STL	**0.0023**	> 0.9999
	vs. F-PNA	**< 0.0001**	**0.0004**
**F-GNL**	vs F-WGA	**< 0.0001**	**0.0008**

	vs F-LCA	0.4948	0.6035
	vs. F-UEA I	0.9967	0.9349
	vs. F-STL	0.0954	0.9859
	vs. F-PNA	**0.0016**	**0.0020**
**F-STL**	vs F-WGA	**< 0.0001**	**0.0009**
	vs. F-LCA	0.0789	0.3398
	vs. F-UEA I	**0.0023**	> 0.9999
	vs. F-GNL	0.0954	0.9859
	vs. F-PNA	0.1430	**0.0010**
**F-PNA**	vs F-WGA	**< 0.0001**	**0.0001**
	vs. F-LCA	**0.0236**	0.0610
	vs. F-UEA I	**< 0.0001**	**0.0004**
	vs. F-GNL	**0.0016**	**0.0020**
	vs. F-STL	0.1430	**0.0010**

Statistical differences were assessed by a Welch's ANOVA test and a Dunnett's T3 multiple comparisons test. P values in bold indicate statistically significant differences between comparisons. Results were considered statistically significant if $p \leq 0.05$. Data presented in Figure 2.

Table S3. Results of uptake studies.

Lectin	**Uptake [%]** (mean ± SD)		**Adjusted P value**	**Uptake [nM]** (mean ± SD)		**Adjusted P value**
**F-WGA**	18.88 ± 9.85	vs. F-LCA	0.5033	47.14 ± 25.03	vs. F-LCA	0.6453
		vs. F-UEA I	0.0930		vs. F-UEA I	0.2721
		vs. F-GNL	0.2774		vs. F-GNL	0.0929
		vs. F-STL	0.3594		vs. F-STL	**<0.0001**
		vs. F-PNA	**< 0.0001**		vs. F-PNA	**0.0249**
**F-LCA**	27.22 ± 8.86	vs F-WGA	0.5033	35.15 ± 13.87	vs F-WGA	0.6453
		vs. F-UEA I	0.9904		vs. F-UEA I	0.9990
		vs. F-GNL	> 0.9999		vs. F-GNL	0.9544
		vs. F-STL	**0.0167**		vs. F-STL	0.0185
		vs. F-PNA	0.0955		vs. F-PNA	0.7601
**F-UEA I**	30.12 ± 8.18	vs F-WGA	0.0930	32.22 ± 8.50	vs F-WGA	0.2721
		vs F-LCA	0.9904		vs F-LCA	0.9990
		vs. F-GNL	0.9939		vs. F-GNL	0.9936

		vs. F-STL	**0.0006**		vs. F-STL	**0.0163**
		vs. F-PNA	0.1789		vs. F-PNA	0.8771
		vs F-WGA	0.2774		vs F-WGA	0.0929
		vs F-LCA	> 0.9999		vs F-LCA	0.9544
**F-GNL**	27.82 ± 9.34	vs. F-UEA I	0.9939	28.45 ± 12.61	vs. F-UEA I	0.9936
		vs. F-STL	**0.0030**		vs. F-STL	0.0639
		vs. F-PNA	0.0555		vs. F-PNA	0.9934
		vs F-WGA	0.3594		vs F-WGA	**<0.0001**
		vs. F-LCA	**0.0167**		vs. F-LCA	**0.0185**
**F-STL**	10.62 ± 5.50	vs. F-UEA I	**0.0006**	8.62 ± 4.47	vs. F-UEA I	**0.0163**
		vs. F-GNL	**0.0030**		vs. F-GNL	0.0639
		vs. F-PNA	**< 0.0001**		vs. F-PNA	0.2038
		vs F-WGA	**< 0.0001**		vs F-WGA	**0.0249**
		vs. F-LCA	0.0955		vs. F-LCA	0.7601
**F-PNA**	40.34 ± 8.52	vs. F-UEA I	0.1789	24.65 ± 7.80	vs. F-UEA I	0.8771
		vs. F-GNL	0.0555		vs. F-GNL	0.9934
		vs. F-STL	**< 0.0001**		vs. F-STL	0.2038

Statistical differences were assessed by a one-way ANOVA test and a Tukey's multiple comparisons test. P values in bold indicate statistically significant differences between comparisons. Results were considered statistically significant if $p \leq 0.05$. Data presented in Figure 5a (Uptake; %) and Figure 5b (Uptake; nm), respectively.

Table S4. Reduction of -mucosa-associated lectins by washing.

Lectin	Reduction [%] (mean ± SD)		Adjusted P value
		vs. F-LCA	0.6136
		vs. F-UEA I	0.5742
**F-WGA**	24.88 ± 6.32	vs. F-GNL	0.8580
		vs. F-STL	0.9905
		vs. F-PNA	0.9722
		vs F-WGA	0.6136
**F-LCA**	16.94 ± 6.97	vs. F-UEA I	**0.0463**
		vs. F-GNL	0.9920

		vs. F-STL	0.3036
		vs. F-PNA	0.2381
**F-UEA I**	32.11 ± 11.97	vs F-WGA	0.5742
		vs F-LCA	**0.0463**
		vs. F-GNL	0.0797
		vs. F-STL	0.8989
		vs. F-PNA	0.9481
**F-GNL**	19.83 ± 7.90	vs F-WGA	0.8580
		vs F-LCA	0.9920
		vs. F-UEA I	0.0797
		vs. F-STL	0.5102
		vs. F-PNA	0.4115
**F-STL**	27.51 ± 6.34	vs F-WGA	0.9905
		vs. F-LCA	0.3036
		vs. F-UEA I	0.8989
		vs. F-GNL	0.5102
		vs. F-PNA	> 0.9999
**F-PNA**	28.23 ± 10.81	vs F-WGA	0.9722
		vs. F-LCA	0.2381
		vs. F-UEA I	0.9481
		vs. F-GNL	0.4115
		vs. F-STL	> 0.9999

Statistical differences were assessed by a one-way ANOVA test and a Tukey's multiple comparisons test. P values in bold indicate statistically significant differences between comparisons. Results were considered statistically significant if $p \leq 0.05$. Data presented in Figure 6.
